# Centering Africa as context and driver for Global Health Ethics: incompleteness, conviviality and the limits of Ubuntu: This article is based on the opening keynote address at the Oxford Global Health and Bioethics Conference, Oxford, June 2023

**DOI:** 10.12688/wellcomeopenres.22508.1

**Published:** 2024-07-10

**Authors:** Jantina de Vries

**Affiliations:** 1 The EthicsLab, Department of Medicine, University of Cape Town, Rondebosch, Western Cape, South Africa; 2 Neuroscience Institute, University of Cape Town, Rondebosch, Western Cape, South Africa

**Keywords:** Global Health Ethics, Africa, dehumanisation, scarcity, incompleteness, conviviality, ethics, ubuntu

## Abstract

Silences exist in global health ethics scholarship because of the particular caricatures of Africa that abound in the world, and these silences profoundly impact scholarship in this field. In this paper, I outline three such silences. The first concerns the consequences of representations of Africa as a place of theoretical scarcity, where the only theory seemingly worth mentioning is relational ontology. The second issue I highlight is the impact of dehumanization on global health and ethics. The third concerns the expectation that African science should serve the goal of development, which limits not only the scientific imagination but also the range of ethical questions that are engaged with. Finally, I turn to Francis Nyamnjoh’s theory of incompleteness and conviviality to propose a shift in bioethics scholarship towards increased focus on the interconnections, encounters and mutual dependency of people and places elsewhere. Incompleteness requires epistemic humility and a curiosity about the views and experiences of others; conviviality is the predisposition required to allow for meaningful exchanges and mutual learning in global health ethics. As a theoretical framework, incompleteness and conviviality are part of a rich African intellectual tradition to help articulate opportunities for a transformative research agenda that helps us understand our world, and its crises, better.

## Plain Language Summary

Global health ethics discussions often ignore important issues because of biased views about Africa. This paper highlights three such overlooked areas. Firstly, Africa is often seen as lacking in theoretical ideas beyond relational ontology. Secondly, dehumanization - or treating people as less-than-human because of where they live, how wealthy they are, or how they look - is often overlooked in global health ethics. Thirdly, there is an expectation for African science to only focus on development, limiting both scientific and ethical exploration. The paper suggests using Francis Nyamnjoh’s theory of incompleteness and conviviality to shift focus towards understanding knowledge as arising from the connections and dependencies between different people and places. This approach encourages humility and curiosity about others' perspectives, fostering meaningful exchanges and mutual learning in global health ethics.

In a paper I wrote with Bridget Pratt, we asked ‘where is knowledge from the global south?’ ^1^. In a response to that paper, Seye Abimbola suggested not only that that was a somewhat silly question to ask, but also that the work that ought to happen to promote epistemic justice in global health ethics, is for people in the periphery to imagine themselves in the centre ^2^. Contributors to decolonial scholarship make similar points. For instance, in a recent paper tracing bird-naming conventions, anthropologist Jess Auerbach from UCT and others use an upside-down map of the world that provocatively challenges scholars from the South to imagine themselves in it centre, not periphery ^3^.

As a scholar based in South Africa, I find the invitation to imagine ourselves at the top of the world – or at least, at its centre – simultaneously alluring and intimidating. What I hope to do in this paper, is to respond to the challenge and explore what it means to deliberately centre Africa as the context and driver for global health ethics.

I want to make a simple but important point: that silences exist in our scholarship because of the particular caricatures of Africa that abound in the world, and that these silences profoundly impact global health ethics scholarship. I will start off with describing three such silences. The first of these concerns the consequences of representations of Africa as a place of theoretical scarcity, where the only theory seemingly worth mentioning is relational ontology. The second issue I highlight will be the impact of dehumanization on global health. The third concerns the locking of African science into developmentalist narratives. I highlight these three issues as problems that require both practical and theoretical attention in the field of global health ethics. I then turn to Francis Nyamnjoh’s work on incompleteness and conviviality as a possible theoretical framework to help reposition and reimagine global health ethics from the South.

Thinking about Africa from the South, the first question that comes to the fore is what are the images or representations of Africa that guide our scholarship and what are the limitations of that purview? There have been ample critiques of popular representations of Africa as a place of scarcity – as a place that is always characterized by drought, famine, violent conflict, disaster and emergency. To speak with the words of Jean-François Bayart, the tiresome representation of Africa as a place of marginalization “does no more than reproduce Hegel's idea that this part of the globe is an 'enclave', existing in 'isolation' on account of its deserts, its forests and its alleged primitiveness” ^4: 217^. Others, like South African philosopher Pascah Mungwini, make similar claims ^5, 6^. Whilst I am not going to repeat those critiques here, I suggest that equally, Africa is often presented as a place of theoretical scarcity as well. I will outline how that is the case in bioethics discourse and the effects it has had on our scholarship.

## Silence I: Africa as a place of theoretical scarcity

On the one hand, the idea of Africa as a place of theoretical scarcity has allowed the perception that Africa is a place where others can come and place, transpose, or impose their own theories and ideas; as a place where others could come – or perhaps should come – to offer concepts and theories about ways of being and about ethics that fill the gap. In fact, understanding Africa as a place of theoretical scarcity has allowed the complete imposition of ideas about ethics from elsewhere, on research contexts and practices on the African continent. In the interests of time, I will ignore the entanglement between 18 ^th^ century Western philosophy and social theory and the political quest for domination which precipitated the colonial era ^7^. But even if I focus my gaze only on the contemporary moment, the 20 ^th^ century expansion of a limited set of ideas and approaches in ethics to the entire world, was obviously also deeply problematic. In particular, the global expansion of principles-based ethics, the hegemony of autonomy and individual rights, as well as the peculiar ways in which bioethicists have – or have not – engaged with questions of social justice, are challenges in the field of bioethics ^8^. The wholesale imposition of ideas and practices in ethics on African research contexts, was, I would propose, a direct consequence of thinking about Africa as a place that was ‘pre-ethical’ ^9^ – as a place of theoretical scarcity. We are now witnessing an equally problematic consequence of thinking about Africa as a place of theoretical scarcity.

In recent years, we have seen scholars starting to take seriously elements from African philosophy in their work in ethics – which certainly is progress of some sort. Specifically, those scholars are trying to understand better how notions of relational ontology, and ideas around Ubuntu, could or should inform how we think about ethics. But two new kinds of problems are emerging.

The first of these is the idea that African philosophy, and ideas around Ubuntu, are settled, stable and uncontested – that they can be packaged as a coherent set of ideas that are universally shared by all those who call themselves African. ‘Ubuntu’ philosophy – or communitarian philosophy, or relational ontology as people also call it – is then presented as quintessentially and universally African; as the way in which people on the African continent live and make sense of life. Recently, we have witnessed a proliferation of academic ethics papers that make use of the term Ubuntu, across a wide range of areas of application including for instance AI ^10^, big data ^11^ and the environment ^12^. Ubuntu has also been called ‘the heart of African ethics’ ^13^ and essential in the quest for decolonizing research methods ^14^. Collectively, the impression is created that the application of African relational philosophy – or ideas about Ubuntu – to discussions about ethics is essential, straightforward and almost formulaic.

The truth is, of course, not that simple. Even between writers who use the term Ubuntu to describe their work, there are contestations and unsettled debates, just as there are contestations and unsettled debates in philosophy generally ^15^. For instance, there is no agreement about the exact status of the individual in community, or about whether non-human organisms, past and future humans or even objects have moral status. Similarly, there are African philosophers and feminists for whom ideas of ‘relational ontology’ or ‘Ubuntu’ explain very little about African ways of being. Bernard Matolino and colleague, for instance, critique the universalizing of ideas (analogous to) Ubuntu to describe the experience of being human on the African continent ^16^; and Nyasha Mboti has criticized definitions of Ubuntu as ‘consistently and purposefully fuzzy’ ^17^. Yet others emphasize the entanglement of accounts of relationality with the politics of decolonization and the project of nation-building ^18^. Furthermore, female African theorists have cautioned that the emphasis on communitarianism has powerfully preserved the conservative patriarchy that continues to silence the voices and perspectives of others ^19^.

The point of the critique here is not to undermine the important scholarship of people who find the framework of Ubuntu and analogous terms helpful as a tool to understand ethics. Rather, the point is to point out that as African ideas gain traction and popularity in global health ethics discourse, complex ideas have been co-opted to mean something stable and complete – translated into something formulaic that can be applied to all kinds of practical problems. Through this co-option, these ideas are made static, and lose their potency to offer alternatives, to critique, and to help us re-imagine the world. Furthermore, the reduction of these ideas to static definitions also seems to reduce their place, history and significance, which in itself is indicative of representations of Africa as a place of theoretical scarcity.

By limiting our gaze in bioethics to thinking about Ubuntu (and analogous terms) as the only concept that defines African intellectual contributions to our scholarship, we miss out on the intellectual opportunities that abound in a space that has always had to define itself through encounters with others ^20, 21^. There is a lineage of African intellectuals who have all drawn on Africa’s and Africans’ mobility and encounters with others as vantagepoints from which to view the world. They have explored movement and flux, relationality and encounters as constantly defining and shaping us. Understood in this way, African scholarship is global. These theorists are comfortable with abandoning ideas about ‘zero sums’ or absolute ideas about human and non-human, African or non-African. They are also comfortable with and have theorized about uncertain futures ^22^. In a world of synthetic embryos and generative AI that effectively mirrors or mimics myriad aspects of human ability, these theories offer wonderful opportunities to consider a future, dispersed human that is profoundly interconnected to embodied or out-of-body technologies. They also offer opportunities to consider, in practice, what a pluriversal approach to ethics could look like and how to think about ethics from the vantagepoint of interconnections between past, present and future peoples, and between people and the natural world. 

Furthermore, African theorists, including Ubuntu philosophers like Motsamai Molefe and feminist theorists like Abena Busia, see in ideas of relational ontology an invitation to ensure that our gaze is firmly on the relations of power, domination and oppression that characterise our modern world. They emphasize, at all times and in all situations, the centrality of questions of human dignity and social justice; on understanding the ways in which our modern world and the technologies that surround us, perpetuate or challenge the existing status quo which favours the few at the cost of many. In a world marked by ever-widening gaps between elites and everyone else, encroaching technology and the destruction of our natural world, African theorists’ engagement with themes of oppression, social justice, and identity through encounters offers powerful opportunities to re-imagine not just our scholarship but the world.

## Silence II: dehumanization

A second question that comes to the fore when thinking about global health ethics from South Africa, relates to the continued invisibilising of the agency – and accountability – of Africans. Building on Eve Tuck’s powerful letter to communities called ‘Suspending Damage’ ^23^, it strikes me that much of global health and ethics research that I encounter, thrives on imaginations of Africans as broken. Such research thrives on pathologizing the experiences of the ‘disenfranchised and disposessed’ ^23: 409^. In global health and global health ethics research, Africans and Africa are curiously both present and absent at the same time; researched yet ironically invisible. Present in the sense that both are the subject of much scholarship, and absent in the sense that the fullness of people’s worldly experience is often missing from our texts. Practical examples that I have encountered over the years include for instance qualitative research on child malnutrition and child death, where grief, shame or resilience of parents seeking to provide for their children is not deemed worth mentioning or investigating; the same is true in projects pursuing techniques for minimally invasive autopsies in children ^24^. In studies of consent, we occasionally mention patriarchy but rarely stop to think how that affects or constrains the experiences of the people in our research – a curious gap at the heart of our scholarship.

Psychologists use the term ‘dehumanization’ to describe the process of depriving a person or population of human qualities or attributes such as independent thought, emotion, compassion, dignity or individuality ^25, 26^. Put simply, the process of dehumanization involves the ‘othering’ of people and it is an essential component of the process of discrimination. We have evidence from around the world that people of colour in general, and people of black African descent in particular, are often dehumanized. For instance, my colleague Melike Fourie and others found that in South Africa, despite significant changes in power dynamics post-apartheid, dehumanization ratings mirrored the apartheid-inspired racial hierarchy, with white people rated “more human” and black African people rated as ‘less human’ across the study ^27^. Levels of dehumanization were associated with reduced support for policies and practices that would promote social equality and justice. In other words – the less people were seen as ‘fully human’, the less support there was for policies and practices that would level the playing field. We know that this painful reality is true the world over: time and again, research on dehumanization showcases that the browner people’s skin colour, or the thinner their wallet, the less people are seen as ‘fully human’ ^25, 28, 29^.

When global health researchers fail to attribute to research participants the fullness of human emotions including pain and grief, we witness the consequences of dehumanization. Importantly, the more rural and poor the subjects of research, the greater this apparent tendency to dehumanize the individuals involved. Combined with scarcity, the focus is invariably on suffering and victimhood; resilience, agency and resourcefulness are ignored.

Why this matters is that dehumanisation does not just happen - it has a function. It is not an accident that there is a correlation between greater dehumanisation and less progressive redistribution policies - part of the function is to maintain the status quo - which is exactly why bioethics must consider this idea seriously. Dehumanisation allows the field of global health to continue to be structured in the way that it is. It allows a structure whereby people from the ‘minority world’ – people who may come from wealthy countries, who may be white, have high levels of educational attainment and share the wealth that comes with a comfortable, middle-class life anywhere in the world – continue to study and consider the lives, beings and ethics of people from the majority world, who may be dehumanized on the grounds of their skin colour, place of origin, or level of wealth.

Dehumanization also features in an entirely different way in global health research. Specifically, just like the agency of research participants is denied, so too is the agency of politicians in Africa. In the eyes of theorists like Jean-François Bayart, there is increasing evidence that the perpetual state of political disaster in many African countries, is a deliberate political act ^4, 30^. In this understanding, African political elites deliberately perpetuate or maintain political chaos within nation states in order to secure access to international funding, which is then embezzled ^31^. In this image, African states are extraverted – oriented towards the outside – rather than introverted, or oriented towards their citizens. This extraversion of nations and governments perpetuates the poor health of citizens of many African countries – which international global health scholars and organisations then try to address. African politicians have agency – even if we don’t see or recognize it. And yet, we do not tend to hold African politicians accountable for their failure to act in the best interests of their citizens. In fact, we are largely silent on the thorny topics of corruption or other abuses of power in global health ethics discourse. Furthermore, as my colleague Caesar Atuire has pointed out ^1^, even at the level of international conventions – such as for instance the new pandemic treaty – the accountability of African politicians is ignored. But how does our collective unwillingness to speak about power and domination, serve to perpetuate the status quo and continue to fail the poorest people in our societies?

## Silence III: science as developmentalism

There is a third and final point I would like to make in the context of my broader quest to position Africa as the driver of global health ethics, and this relates to a collective failure to recognize the way in which discourses on science and innovation in the African continent are locked into global illusions of developmentalism. On the one hand, there is an illusion that the best science and innovation originates in places with stable democracies, moderate climates and stable infrastructure. In that model, African science and innovation are always second-best, and African scientists always need to play catch-up or to translate or make relevant their work in relation to work from dominant places. On the other hand, there is a strong belief that for science to happen on the continent, it needs to promote development. In this rhetoric, Western development is the blueprint for global development that science elsewhere ought to promote. This means that in order for African science to be considered meaningful, it needs to demonstrate both how it mimics Western science, but also how it promotes ideals of development, marketisation, privatization, and individual health. Several years ago, Zambian economist Dambisa Moyo wrote a powerful critique of the stifling effect of similar rhetoric on African economic development; I think we need to reflect much more deeply on the stranglehold that these dual illusions have on science, technology development and innovation on the African continent.

## Incompleteness and conviviality

I started this article aiming to explore what it would mean to consider ‘Africa’ as context and driver in global health ethics. I developed three critiques of global health ethics which were the implications of considering Africa as a place of theoretical scarcity, the reality of dehumanization of African participants and politicians, and the locking in of African science and innovation in developmentalist discourses. The second part of this article outlines a predisposition worth cultivating that grounds how we approach this work: recognising and providing for incompleteness.

Much of my thinking about the transition that happens when we think about Africa as the driver for global health ethics, is framed in terms of UCT Anthropology professor Francis Nyamnjoh’s theory of incompleteness and conviviality ^32^. Nyamnjoh defines incompleteness as “a framework for thinking, articulating, relating to and understanding a world in which to be incomplete is normal and universal. Incompleteness touches on all aspects of existence or being and becoming at individual as well as collective levels, and applies to humans and their relationships with non-humans, natural and artificial, animate and inanimate” (Ibid: pg15). Nyamnjoh’s theory proposes an alternative to Cartesian dualisms that divide the world into categories like African and Western, modern and traditional, human and non-human – the same dualisms that drove the colonial conquest. Responding to damage that such thinking has done to knowledge production and to our modern world, Nyamnjoh proposes that the more fluid, dynamic and inclusive ways of knowing the world characteristic to African representations of personhood and agency can rejuvenate our scholarship. Importantly also, it can provide the contours of a scholarship that is not entrapped in the same kind of essentialisms that supported the colonial conquest of Africa.

One theoretical opportunity in Nyamnjoh’s account lies in his reminder that our fantasies of completeness in terms of what makes a fully human – fantasies of whiteness, of particular kinds of political and economic freedom and achievement and of individuality – causes us to recognize others as only partly human. The further they are removed from the ideal-type, the less fully their human experience is recognized in global health ethics. Another opportunity is inherent to the invitation to consider knowledge as always arising out of the encounters – as neither Western or African, but co-constituted by the experiences, knowledge and context of both places and ways of being. Finally, Nyamnjoh’s idea of incompleteness helps us understand the world as interconnected through space and time – and mutually dependent. It draws attention to the fact that ideas of – and opportunities for – ‘development’ are critically connected to and dependent on the underdevelopment of places elsewhere; that the power and privilege that allows some universities to thrive is not disconnected to the precarity of science and societies elsewhere.

But perhaps more powerfully than all of that, for Nyamnjoh the acceptance of incompleteness is an invitation to celebrate the extent to which our beings and knowledge are a permanent work in progress. It is in this sense that I draw on his work: as an invitation to observe the way in which our view of the world is partial or incomplete, and the way in which fantasies or delusions of completeness have informed our thinking about, and being in, the world. And with that insight, we then have an opportunity to course correct: to curiously engage with what happens when we rather embrace incompleteness in terms of our theoretical orientation, our knowledge or our worldly experience.

Again, speaking with his words, not only does this require humility and courage to embrace incompleteness, but also that “Humility and the modesty it suggests, inspires the cultivation of an enduring capacity to rise above an attachment to self, the obsession with being right, and taking one’s self too seriously” (ibid pg 33). Letting go of those ideas opens up the possibility of conviviality: a predisposition of curiosity with the experiences and views of others, an interest in their stories, and a recognition of the way in which we are all constant work in process.

This work involves everyone, but not in a way that bends “backwards … to accommodate those determined to play zero-sum games of conquest or … continue to act with callous indifference to the predicaments of the subjected. Convivial scholarship is a scholarship for naming and shaming impunities, its violence and violations” (Ibid pg 33). It involves a continuous excavating of “the hierarchies and silences in knowledge production spheres in the academy, in a way that shines the torchlight on the epistemic and related injustices that have come with the brutality, violence and violations of the colonial order”. It is in this sense that I draw on incompleteness and conviviality.

Which brings me to the conclusion of this article, which is the invitation to people whose scholarship is in the discipline of bioethics and whose work is relevant to the African continent, to be much bolder and imaginative in their engagement with Africa both as a geographical space but also as a source of exciting and original scholarship that can help us understand better what it means to be human. Scholarship that for decades has grappled with complex ideas about liberation ^33^ and transformation and scholarship that cannot and should not be flattened to formulaic ideals of an Ubuntu ethics. A bioethics scholarship that engages a rich African intellectual tradition to help articulate opportunities for a transformative research agenda that helps us understand our world, and its crises, better.

## Disclaimer

The views expressed in this article are those of the author. Publication in Wellcome Open Research does not imply endorsement by Wellcome.

## Data Availability

No data are associated with this article.
