# Supplementary material for: Centering Africa as context and driver for Global Health Ethics: incompleteness, conviviality and the limits of Ubuntu: This article is based on the opening keynote address at the Oxford Global Health and Bioethics Conference, Oxford, June 2023
Source: Wellcome Open Res. 2024 Jul 10;9:371. [Version 1] doi: 10.12688/wellcomeopenres.22508.1 (PMC12504929; doi:10.12688/wellcomeopenres.22508.1)
Supplement: Peer review history

# Reviewer report: Reviewer response for version 1

**DOI:** 10.21956/wellcomeopenres.24800.r133975

**Published:** 2025-10-31

**Authors:** Alberto Giubilini, Tolulope Osayomi

**Affiliations:** 1 University of Oxford, Oxford, UK; 2 Geography, University of Ibadan Department of Geography, Ibadan, Nigeria

How can Africa be the context and driver of global health ethics? This is the question de Vries’s article tries to answer. She argues that Africa has historically been at the margins of the global knowledge production enterprise not because it lacks the capacity for significant scholarly work, but because of the several negative stereotypes or misrepresentations of African scholarship. She nicely describes them as “silences” around Africa. The critique of these silences represents the main target of de Vries’ article. Analyzing these stereotypes can contribute to a better understanding of Africa’s unique and plural cultural identity and, consequently, of its potential, pluriversal contribution to discussion in global health ethics.

 The article claims the status of driver of global health for an ethics of incompleteness and conviviality, which has its roots in the African cultural and intellectual milieu. It would be helpful if an article with this aim said something about how different perspectives originating in different cultural contexts could be integrated into an overarching account that could be meaningfully understood as ‘global’ in scope [see e.g. (Giubilini, 2025 - ref 1) for a challenge to this enterprise].

 Doing that might help De Vries address a question that her analysis seems to raise, but that is unanswered in her article. That is, why is the author particularly interested in the specific framework of incompleteness and conviviality, among the many that could express an African perspective and a potential contribution to global health ethics? There are other equally unique frameworks that are potentially valuable to the discourse, such as the Ọmọlúàbí philosophy and Àsùwádà epistemology based on the Yoruba values of solidarity and interdependence (Akiwowo,1986) and many more.  Restricting the discussion to just one theoretical framework would largely defeat the spirit and purpose of plurality which she proudly promotes. This could have been a great opportunity for the appreciation of the rich and truly diverse African intellectual traditions.

 Besides, just as the Western theories have their strengths and limitations, so do indigenous theories. We assume the theory of incompleteness and conviviality has its deficiencies of its own that might be in tension with the aspiration of ‘driver’ of global health ethics. After all, if incompleteness is taken to be the normal condition of human life and human thinking , the framework could be ‘incomplete’ itself.

 de Vries’ focus on the silences around Africa and Africa’s scholarship is insightful and opens up a much-needed space for discussion, quite regardless of whether and how that squares with the ‘global health’ terminology. She identifies three types of silences:

**Silence 1: Africa as a place of theoretical scarcity:** This statement, in our opinion, is a mild description of an epistemic injustice. The truth is Africa, until recently, has been viewed for long as a theoretically poor continent and therefore incapable of theorization and theory driven research. This significantly explains why Africa appears to be a wholesale consumer of foreign epistemes, concepts and theories as well as a testing ground for theories essentially built in and for foreign contexts.

 We need to promptly stress that this is in no way an outright rejection of foreign theories and concepts. As just some of them have been found to be useful, epistemologies of the global south are equally valuable. An example, as mentioned multiple times, is the Ubuntu philosophy. Its adoption signifies the need for centring African beliefs and values in bioethics. This of course could open the flood gate for many more global south epistemologies of great potential, which in the end  creates  a climate of pluriversality.

**Silence 2: Dehumanisation**

 De Vries may not have realized that the first silence informs the second. They are apparently “two sides of a coin”. They are inextricably linked. The second silence touches on the invisibility or perhaps absence of African agency. The popular opinion is that African societies lack the creative agency to shape outcomes in their favour.  Here, De Vries brilliantly presents a paradox in Africa’s reality- the world seeing Africans as ‘present yet absent’ – “present in the sense that both are subject of much scholarship and absent in the sense that their lived experiences are absent”. This reechoes (Mignolo, 2009 - ref 3)’s critical observation on Africa being either the data ****source or just being the data itself. To put it differently, Africa and Africans are ‘researched yet unseen or unrecognised’.  Some scholars in their publication: *Who is a Global Health Expert?* noted with displeasure that LMIC authors are excluded from the knowledge production process despite the fact they had both the expertise and lived experiences. (Ojiako et al 2023). 

 This othering is, in an important sense, an act of dehumanization. It creates situations in which some people are either more or less worthy of consideration than others.  Moreover, it simply seeks to amplify the weaknesses rather than the strengths of Africans, thereby reinforcing the prevailing racial hierarchy in the knowledge production process. This takes our mind back to an incident during the COVID-19 pandemic in Africa. A BBC opinion piece published in 2020 with the controversial question “Could be poverty a vaccine against COVID-19?” drew a large-scale public outcry from African countries and the diaspora. This was, as one would have expected, not taken lightly. In fact, one fondly remembers an online reaction on X: “ *They said we will die because we are poor, and now they are saying we are not dying because we are poor*.”

 Candidly speaking, it was a rather curious response to the lower-than-anticipated COVID-19 mortality rates in the continent. However, a more thorough analysis of this claim suggested that there was no association between COVID-19 and poverty in Africa (Osayomi et al. 2021 - ref 5). This alleged “African COVID-19 Paradox”, as it has come to be known, exposes the failure or perhaps the refusal of the outside world to accept a counter story of Africa and recognise, and more importantly celebrate its ingenuity and resourcefulness.

 Following Paul Farmer’s famous quote “ [t]the idea that some lives matter less is the root of all that is wrong of all that is wrong with the world”, we can firmly say that there is no honour in denying the dignity and identity of a people.

**Silence 3: Science as Developmentalism**

 The last silence, Science as Developmentalism, is a recognition of the hegemony of science in development narratives. Science is superficially portrayed as the pathway for socioeconomic progress and gold standard for societal advancement.  In de Vries’ words, “best sciences and innovation originate in places with stable democracies, moderate climates and stable infrastructures”. Hence, if African societies must achieve the progress they desire, they must adopt the Western template/blueprint for African development.  In remarks like this lies the colonial matrix of power. It describes how coloniality shapes our existence. According to (Naidu, 2024 - ref 4), the Western and Eurocentric perspectives are superior to others.

 Therefore, de Vries calls for a level playing field, opening up space for alternative and indigenous thought. In a similar vein, an urban and development geographer,  (Mabogunje, 2015 - ref 2) in 1977, plainly expressed “the need to re-examine our present strategies of development in order to determine how they are suitable, adequate and appropriate for the task of developing our society” and thus  recommended Africans “…accept the non-viability of this course of action and to settle down to fashioning new developmental goals and strategies more consistent with their local resource endowment and socio-cultural disposition” (p.27).

 In our view, this is the appropriate approach to adopt. Upholding this silence would be absolutely reductionist and unjust.

 In conclusion, we see that the author expresses reservations with the romanticization of the Ubuntu philosophy, suggesting integrating it with Francis Nyajmjoh ‘s theory of incompleteness and conviviality. The canon of this theory is the fact that “incompleteness is normal and universal”.  No one and no condition can attain a perfect state. We are not whole in ourselves; hence we need interdependence to survive.  It is an exciting theory that reminds of two similar global south perspectives. The first is the Chinese philosophy of Ying-Yang. It underscores the essence of complementarity in human relations. The second perspective is the Yoruba worldview based on this verse from Odu Ifa (corpus of Ifa, the Yoruba god of divination), emphasizing the significance of cooperation and interconnectedness in life):

 “Àṣọ fúnfun ṣùnkùn àró, ìpìlẹ̀ òrò ńṣùnkùn ìkejì tàntàntàn

 (‘The white cloth cries out for the indigo dye just as the first remark impatiently awaits the next’)

Does the article adequately reference differing views and opinions?

Yes

Are all factual statements correct, and are statements and arguments made adequately supported by citations?

Yes

Is the Open Letter written in accessible language?

Yes

Where applicable, are recommendations and next steps explained clearly for others to follow?

Yes

Is the rationale for the Open Letter provided in sufficient detail?

Yes

Reviewer Expertise:

for Alberto Giubilini: Philosophy, Bioethics, Public Health Ethics; for Tolulope Osayomi: Medical Geography

We confirm that we have read this submission and believe that we have an appropriate level of expertise to confirm that it is of an acceptable scientific standard, however we have significant reservations, as outlined above.

## References

[1] : What in the world is global health? A conceptual analysis. *Developing World Bioethics* .2025; 10.1111/dewb.12478 10.1111/dewb.12478 PMC761831739989230

[2] : The Development Process.2015; 10.4324/9781315658483 10.4324/9781315658483

[3] : Epistemic Disobedience, Independent Thought and Decolonial Freedom. *Theory, Culture & Society* .2009;26(7-8) : 10.1177/0263276409349275 159-181 10.1177/0263276409349275

[4] : The personal is political in the struggle for equity in global medical education research and scholarship. *Medical Teacher* .2023;45(9) : 10.1080/0142159X.2023.2206535 991-996 10.1080/0142159X.2023.2206535 37200518

[5] : A Geographical Analysis of the African COVID-19 Paradox: Putting the Poverty-as-a-Vaccine Hypothesis to the Test. *Earth Systems and Environment* .2021;5(3) : 10.1007/s41748-021-00234-5 799-810 10.1007/s41748-021-00234-5 PMC820078434723080

# Reviewer report: Reviewer response for version 1

**DOI:** 10.21956/wellcomeopenres.24800.r133972

**Published:** 2025-10-10

**Authors:** Fayemi Ademola

**Affiliations:** 1 Australia Catholic University, Queensland Bioethics Centre, Brisbane, Australia

Convinced about the nature of our being and knowledge as a work in progress through encounters, a theory of incompleteness and conviviality by Francis Nyamnjoh, which Jantina de Vries (2024) draws on in her article, I accept the privileged invitation to review her article. It is an opportunity to contribute to the emerging conversational space of proposing, contesting, improving, and deepening encounters of ideas from and across global health ethics actors, resulting in competing traditions of thought in global health ethics. 

 In "Centering Africa as context and driver for Global Health Ethics: incompleteness, conviviality and the limits of Ubuntu," de Vries critiques three different silences in global health ethics, which, according to her, are responsible for the marginalisation of African voices in global health ethics and bioethics scholarship. The first silence is the perception of Africa as a place of theoretical scarcity, where relational ontology, especially Ubuntu, seemingly assumes a universally representative image of African ethics and is applied in a formulaic manner to almost every social and practical problem. The second silence is dehumanisation, understood as the practice of making fundamental existential features of a person or people, including agency, accountability, dignity, and independent thought, invisible and deprivative. The third silence construes science as developmentalism with some restrictive expectations of African science to mimic Western models and serve developmental goals.

 In a very impressive way, de Vries engages with Francis Nyamnjoh's theory of incompleteness and conviviality. Incompleteness is a framework for understanding human nature and knowledge as universally incomplete by default, thereby making room for improvement through encounters with others. Incompleteness overlaps with conviviality, which speaks to the curious epistemic dispositions of engaging with the experiences, views, and stories of others; respecting their contexts and recognising the dependent connections through space and time across experiences and encounters. de Vries excellently linked the three identified silences and their impacts on global health ethics scholarship, proposing Nyamnjoh's theory of incompleteness and conviviality as a corrective. The author emphasises the urgent need to de-silence existing silences in global health ethics by centering Africa as the context and African bioethics scholarship as the driver of global health ethics. 

 A critique of the three identified silences in global health ethics might benefit from an understanding of what global health ethics is. Although the meaning, scope, and goals of global health ethics remain contentious, a clear working definition is lacking in the article. Yet, it is essential for framing the context of the article's subject matter. 

 de Vries made a critical point about the dominance of relational ontology in Africa, with Ubuntu scholarship at the forefront of what seems to be African responses to Western normative theories. Today, Ubuntu is touted as the solution to various social, political, economic, cultural, and environmental challenges, among others, through multiple versions that cater to specific scholarly projects. However, not only does this obscure the creative horizon of ethics knowledge production in the continent, it also seems to occlude the fact of the continent's cultural, moral, and epistemic plurality, with the (possibly unintended) portrayal of Africa as a barren land of ideas and innovative theories.

 de Vries's claim about theoretical scarcity, understood as the limited production of ethics knowledge from Africa and of African pedigree, relative to the proclivity of ethics scholarship elsewhere, deserves some comment. While agreeing with de Vries's call that African ethics scholarship must transcend its current focus on relational ontology, the existence of other African ethical perspectives not grounded in a relational ontology suggests theoretical suppression rather than theoretical scarcity. Theoretical suppression is more a function of historical and structural problems, such as dependency on Western ethical canons in postcolonial Africa due to coloniality, poor funding of knowledge production and dissemination platforms, epistemic-politics on what counts as a theory, and systemic invisibility of cognate ethics concepts and categories, rather than a consequence of limited ethics scholarship by Africans and Africanists. Unless these systemic issues are addressed, the call to centre Africa as context and driver of global health ethics may end up as another rhetoric. Yet, de Vries's point on recognising silent voices is instructive as categories and concepts in African ethics, including personhood, communal well-being, justice, reasonableness, dignity, solidarity, and harmony, can profoundly reshape, impact, and expand the theoretical registers of global health ethics/frameworks.  

 Additionally, de Vries's point about dehumanizing scholarship in Africa is particularly instructive. One may, however, wish to ask whether what the author described as the dehumanization of the African entirely agrees with the definition of dehumanization provided in the article. The author perhaps conflates the actual dehumanization of Africans with the narrative on Africans as perpetually dehumanized. While both may share some features, such as the condescending motive of the actors involved, they are not necessarily the same. That Africans are perpetually portrayed as subjects of what Achile Mbembe (2020) calls necropolitics-subjugation of life to the power of death-in Western scholarship (which, according to the author, is not representative of the complexity of their experiences) is different from the fact that African leaders (according to the author) exercise their agency to dehumanize Africans for personal benefits.

 Following the above, a subtle contradiction exists in the articulation of this dehumanization claim, which I do not find irresolvable. On the one hand, the author bemoans the apparently exaggerated focus on the plight of Africans as a people perpetually in suffering, but on the other, also says that the agency of African leaders in bringing about the suffering and dehumanization does not receive sufficient attention. Thus, we have here two simultaneous yet contradictory claims: Africans are excessively portrayed as dehumanized, but at the same time, the agency and complicity of political leadership in the dehumanization of the same people suffer from inadequate attention in global health ethics discourse. The argument is not very strong in its present state.

 Surely, authors and editors must be aware that political leadership is in many ways responsible for the social conditions of any society. This does not, however, always compel excessive focus on the failure of leadership in global health ethics discourse. It is enough oftentimes (not at all times) to mention this important challenge, backed with one or a few citations, while proceeding to the core of the work (except where solutions explicitly lie in political actions). There are numerous works in Western scholarship (not necessarily in bioethics or global health discourse) that focus on the crisis of leadership in Africa. The author should also demonstrate that this bias is peculiar to Africa and is intended to achieve specific goals. For example, in discussing the issue of (global)health inequity among marginalised populations such as asylum seekers in the US or war-ridden Gaza, should global health ethicists focus intensely on the politics of President Donald Trump and the Republican Party, as they both seek to defund the affordable healthcare program without any substantive replacement? While one is not suggesting that ethics scholarship in Africa must mirror Western ethics scholarship, the author's missed opportunity to provide examples of how Western scholars situate ethical issues in transnational healthcare discourse within the agency of political actors in their societies raises a gentle concern. While obscuring such analysis under the label of dehumanization in Africa, it makes one wonder whether this is another subtle instance of an internal act of silencing within global health ethics scholarship, more so, a targeted one, as portrayed.

 In four different places in the article, the author restates the article's goal: to position Africa as the context and driver of global health ethics discourse. This objective, stated this way, is at odds with the notion of incompleteness and conviviality upon which the author's proposed remedy lies. Putting Africa as "driver", in the manner stated and emphasized, suggests more of an effort to achieve a reversal of roles between the current drivers and their passenger, rather than making everyone co-passengers and co-drivers in ways that portray humility and the acceptance of limitations (incompleteness) on the one hand, and complementary capacity (conviviality) on the other. Inverted asymmetry undermines the concept of shared solidarity envisioned in the article.

 Notably, the author also decried the developmentalist agenda that has been foisted on bioethical scholarship in Africa. This section of the paper is very short and unclear. Notably, there is no single citation to support the claims made, which themselves require further elucidation. Who are the people setting this agenda? For whom do they set it in African ethics scholarship? Does the author agree with the bifurcated notion of Western science and African science? What universal attributes make two distinct knowledge claims scientific yet African and Western? Origin? Research location? Applicability? What is intrinsically wrong about orienting research towards development, whether in Africa or elsewhere? Although the paper critiques developmentalist narratives and calls for epistemic justice, it stops short of offering concrete, practical recommendations.  Nevertheless, the article's support for epistemic justice in global health ethics is exemplary.

 To close, addressing questions of how global health research institutions and global health ethics centres can restructure funding, authorship, or ethics frameworks and guidance development to center African perspectives could strengthen the paper further. This is not too much to expect from a paper that critiques the silences in global health ethics that are divorced from the implications of biased views about Africa, politics, and practical life on the continent. However, this article's ultimate strength lies in its successful deepening of conversations about entrenched assumptions in global health ethics scholarship. It provides a clear call for epistemic humility and a convivial framework of scholarly engagement rooted in diverse African intellectual traditions. Such a position consolidates trends in African studies scholarship that argue for a pluriversally modern yet indigenous perspective on the continent, as opposed to a fixation on ideas from imagined idyllic eras (Oyekan, 2021; Atuire 2023).

 Atuire, C. A. (2023). Some barriers to knowledge from the global south: commentary to Pratt and de Vries. Journal of medical ethics, 49(5), 335-336. de Vries J. (2024)

 The Contemporary Africanist Philosopher and the Need for Indigenous Modern Knowledge. Nokoko, 9, 1-18.

Does the article adequately reference differing views and opinions?

Yes

Are all factual statements correct, and are statements and arguments made adequately supported by citations?

Yes

Is the Open Letter written in accessible language?

Yes

Where applicable, are recommendations and next steps explained clearly for others to follow?

Partly

Is the rationale for the Open Letter provided in sufficient detail?

Yes

Reviewer Expertise:

Dr. Ademola Kazeem Fayemi’s areas of competency and interest include Bioethics,  Research Ethics, Global Health Ethics, and African philosophy.

I confirm that I have read this submission and believe that I have an appropriate level of expertise to confirm that it is of an acceptable scientific standard.

# Reviewer report: Reviewer response for version 1

**DOI:** 10.21956/wellcomeopenres.24800.r133977

**Published:** 2025-10-07

**Authors:** Adewunmi Akingbola

**Affiliations:** 1 University of Cambridge, Cambridge, UK

In this thoughtful open letter, Jantina de Vries challenges the persistent marginalization of African scholarship within global health ethics. She identifies three “silences” that continue to shape the field:

1. The portrayal of Africa as a place of theoretical scarcity, where African philosophy is reduced almost entirely to the concept of *Ubuntu*, neglects the continent’s wider intellectual diversity.
2. The dehumanization of Africans, both in research, where African participants’ agency and emotions are minimized, and in governance, where African leaders’ accountability is rarely discussed.
3. The confinement of African science to developmentalism, the expectation that African research must serve Western-defined ideas of “development,” leaving little room for theoretical innovation.

De Vries draws on Francis Nyamnjoh’s theory of incompleteness and conviviality, which views knowledge as co-created, fluid, and mutually dependent. She argues that embracing incompleteness demands epistemic humility, curiosity, and courage to acknowledge that no worldview is ever complete. Conviviality, meanwhile, calls for meaningful engagement and exchange across boundaries.

 Ultimately, she invites scholars to “center Africa”, not merely as a study site but as an intellectual driver of global health ethics, and to move beyond formulaic applications of *Ubuntu* towards a more dynamic and pluralistic African ethical imagination.

 Areas for Improvement.

1. Clarify the conceptual scope of “global health ethics.”
  - *Issue:* The paper assumes familiarity with “global health ethics” without defining its conceptual boundaries.
  - *Actionable comment:* Add a short framing paragraph in the introduction that situates *global health ethics within* broader debates on global health, bioethics, and social justice. For instance, cite relevant papers to illustrate how global health ethics grapples with power asymmetries and contextual ethics. This will anchor your argument and make the paper more accessible to interdisciplinary readers.
2. Nuance the phrase “centering Africa” and avoid the “top of the world” imagery.
  - *Issue:* The metaphor of Africa being “at the top of the world” may inadvertently suggest a reversal of hierarchies rather than an epistemic rebalancing.
  - *Actionable comment:* Rephrase this section to emphasize *epistemic equity* instead of *positional dominance*. For example: “To center Africa is not to displace others from the core, but to create a pluriversal space where multiple epistemologies coexist and inform one another.”
3. Deepen the connection between Ubuntu and Nyamnjoh’s concepts.
  - *Issue:* The argument risks overstating a disconnect between *Ubuntu* and Nyamnjoh’s *incompleteness* and *conviviality*. Several African scholars view these as complementary rather than conflicting ideas.
  - *Actionable comment:* Add a bridging paragraph acknowledging that incompleteness and conviviality can be interpreted as dynamic extensions of Ubuntu philosophy. Cite feminist or ecological interpretations of Ubuntu (e.g., Busia, Etieyibo) to demonstrate this continuity. This would strengthen your argument against formulaic readings of Ubuntu while avoiding the perception that you are dismissing it entirely.
4. Provide concrete illustrations of how “incompleteness” and “conviviality” could reshape practice.
  - *Issue:* The theoretical argument is strong, but the practical application remains abstract. Readers may struggle to see how this framework could inform real-world research ethics or policy.
  - *Actionable comment:* Include short case examples, for instance, how adopting “incompleteness” might change informed consent processes, capacity-building partnerships, or authorship norms in multi-country research. This would demonstrate the translational value of your framework.
5. Integrate the three “silences” more explicitly as interlinked phenomena.
  - *Issue:* The three silences are presented as separate categories, but they conceptually reinforce one another.
  - *Actionable comment:* Add a synthesizing paragraph (either in the transition to the “Incompleteness and Conviviality” section or in the conclusion) explaining how theoretical scarcity, dehumanization, and developmentalism form a cyclical system of epistemic inequality. This integrative framing will give the argument stronger coherence.
6. Engage more explicitly with feminist and intersectional African scholarship.
  - *Issue:* While feminist caution against patriarchy in Ubuntu is mentioned, the discussion does not fully engage with gendered perspectives on epistemic justice.
  - *Actionable comment:* Briefly reference African feminist ethicists such as Nkiru Nzegwu, Pumla Gqola, or Sylvia Tamale to show that relational ethics can also accommodate feminist readings of incompleteness. This would diversify the intellectual lineage and reinforce your call for epistemic plurality.
7. Strengthen the conclusion by articulating next steps for scholarship and praxis.
  - *Issue:* The conclusion eloquently restates the argument but lacks forward-looking guidance.
  - *Actionable comment:* End with 2–3 practical directions e.g., (a) developing curricula that teach African ethical frameworks, (b) supporting South–South research collaborations, or (c) establishing ethical review models that reflect incompleteness and conviviality. These recommendations will give the paper a more action-oriented finish.

Does the article adequately reference differing views and opinions?

Yes

Are all factual statements correct, and are statements and arguments made adequately supported by citations?

Yes

Is the Open Letter written in accessible language?

Yes

Where applicable, are recommendations and next steps explained clearly for others to follow?

Yes

Is the rationale for the Open Letter provided in sufficient detail?

Yes

Reviewer Expertise:

Global health, Public health, Infectious diseases, Epidemiology

I confirm that I have read this submission and believe that I have an appropriate level of expertise to confirm that it is of an acceptable scientific standard.

# Reviewer report: Reviewer response for version 1

**DOI:** 10.21956/wellcomeopenres.24800.r90929

**Published:** 2024-08-21

**Authors:** Eunice Kamaara

**Affiliations:** 1 Moi University, Eldoret, Uasin Gishu County, Kenya

**Beyond centering Africa as context and driver for global health ethics**

- The concept of global health is widely embraced and appropriated especially among international (non) governmental organizations and is now commonly used to refer to a wide range of ideas and practices. Yet, efforts to (re)define the concept continues 15 years after Koplan *et al *(1) came up with, arguably, the most commonly cited definition.  A systematic review and thematic analysis of literature on the definition of global health by Salm *et al* (2) suggests that 34 or more definitions have been recorded after Koplan *et al* published theirs. Koplan *et al *(1) found it necessary to distinguish global health from ‘international health’ and from ‘public health’. 

**This distinction is encapsulated in the terse definition: **

- "Global health is an area of study, research and practice that places a priority on improving health and achieving equity in health for all people worldwide. Global health emphasizes transnational health issues, determinants and solutions, involves many disciplines within and beyond the health sciences and promotes interdisciplinary collaboration and is a synthesis of population-based prevention with individual-level clinical care" (1)
- Against this backdrop, de Vries firmly argues for ‘centering Africa as context and driver for global health ethics’ (3). De Vries’ problematizes the situation of global health ethics scholarship in Africa summing it up in three silences: i) presentation of ‘Africa as a place of theoretical scarcity’; ii) dehumanization of Africans; and, iii) poor regard of African science and innovation and its measure against western developmentalism. Thankfully, de Vries does not stop at diagnosis but goes ahead to prescribe a remedy to the situation using Nyamjoh’s concepts of incompleteness and conviviality (4). I have nothing but praise and admiration of de Vries for clarity of thought and effective presentation of the key arguments. De Vries addresses global ethics scholarship in Africa in simple terms without simplifying the complex subject. But one question lingers: what exactly is global health ethics?  
- In the article under review, rather than begin, at the very least, with an acknowledgment of the complexity of the terms ‘global health’ and ‘global health ethics’, de Vries (3) dives straight into the subject of interest: ‘what it means to deliberately centre Africa as the context and driver for global health ethics”.  While this may be understood because de Vries has made the point on the complexity of global health ethics in her earlier works especially on genomic research ethics (5), to some readers who would wish more attention paid to this background information, de Vries may be seen to be perpetuating the fundamental silence on which the silences that she writes about are founded– silence on the ethics of politics and economics of naming and ‘originating’. Certainly, originating and naming social phenomenon amounts to providing the phenomenon with shape and substance. It is therefore an exercise of power.
- Global health ethics is an entanglement – complex and multifaceted in both conceptualization and application. As various scholars (including de Vries in the article under review) have pointed out, a definition of the concept is not only riddled with controversy but also with critical ethical questions (6,7,8). Context matters. Even as de Vries refers to the need to find alternatives to principle-based ethics, if ethics principles were to be considered universal, their understanding, interpretation, and application vary from one place and time to another.
- With time and geography, issues of cultural diversity; historical socio-economics and politics; as well as issues of history of science and technology, among others emerge.  In turn, these issues of contexts have ethical implications on global health ethics. Inescapably, both terms ‘global health’ and ‘global health ethics’ have imperial origins, scholarship continues to be dominated by high income countries, and therefore, there is need for these to be decolonized (9,10,11,12,13). At the bare minimum, acknowledging the challenge inherent in the very term ‘global health ethics’ would be a natural starting point of any conversation on global health or global health ethics research.

**However, de Vries is spot on - and probably this is the author’s way of making the same point we make in the previous sentence-:**

- "In global health and global health ethics research, Africans and Africa are curiously both present and absent at the same time; researched yet ironically invisible. Present in the sense that both are the subject of much scholarship, and absent in the sense that the fullness of people’s worldly experience is often missing from our texts". (3)
- While we appreciate where de Vries is coming from, we are concerned with appropriation of the word ‘centre’ and worse, the introduction of the term ‘top’.  Should African scholars seek to be “at the top of the world – or at least, at its centre”? How would aspirations to be at the top or at the centre relate effectively with Nyamjoh’s concepts of incompleteness and conviviality? This question reminds us of a statement by Desmond Tutu, then President of the All Africa Conference of Churches (AACC), in a sermon at the 25 ^th^ Assembly of AACC in Nairobi in 1989: “If detention without trial is evil in South Africa, it must be anywhere else’ (14) He was condemning the practice of detention without trial by the government of then President of Kenya, Daniel arap Moi. It also reminds us of an analogy of a circle which the ‘Circle [1], an association of women theologians in Africa, uses to remind everyone that authentic human efforts to understand our world, calls for centering, an endless process in which the centre is not necessarily the opposite of the periphery.  
- Certainly. There is need to question the dominance of western science and innovation as development while dismissing African science and innovation as secondhand, irrelevant, o and at worst, anti-development.  This is in spite of increasing evidence that a neoliberal capitalist model of development, characterized by materialism, consumerism and individualism, is unsustainable and disastrous. However, such questioning should seek not to antagonize but to moderate and harmonize perspectives in line with Nyamnjoh’s theory. As Nyamnjoh proposes, we ought to seek alternatives to Cartesian dualistic thinking which was and continues to be the basis of colonialism of all forms.
- De Vries present the three silences as different issues. However, while this is important for clarity’s sake, it would help to mention even in passing that they all blend into one. A symbiotic relationship exists between the three silences in that any one of them leads to the other and vice versa. For example, Africans are dehumanized in order to make plausible the claim that Africa is a place of theoretical scarcity and therefore Africa has nothing to contribute to development- limitedly understood.  This understanding is key because it emphasizes the need for holistic perspectives to reality which would only be realized with both attitude and practice of incompleteness and conviviality.
- Absolutely. African perspectives to global health ethics “should not be flattened to formulaic ideals of an Ubuntu ethics”.

**We agree with de Vries that Ubuntu ethics made static:**

- "… loses its potency to offer alternatives, to critique, and to help us re-imagine the world. Furthermore, the reduction of these ideas to static definitions also seems to reduce their place, history and significance, which in itself is indicative of representations of Africa as a place of theoretical scarcity". (3)
- Yet, it is plausible to consider incompleteness and conviviality as aspects of Ubuntu. If, according to Ubuntu, personhood is defined only in community, does this not suggest that incompleteness and conviviality are core elements of being human? Is Ubuntu not about creating worthy and peaceful harmonious relationships? While I agree with de Vries that engagement with global health ethics scholarship in Africa is in dire need of deepening, I wouldn’t consider focus on Ubuntu as limited or oversimplification of African theoretical perspectives because incompleteness and conviviality do not contradict Ubuntu. It may be that Ubuntu is at the core of any discourse on African perspectives to ethics in general and specifically global health ethics. 
- Deepening discourse on Ubuntu may bring to the surface specific African theoretical perspectives – especially given the African concept of an ethical community that does not limit relationships to among humans but relationships with everything that exists including the so called ‘non-living’ realities. Perhaps, as is my view, incompleteness and conviviality may be considered as expositions of Ubuntu. Certainly. We need to engage profoundly with African perspectives in a spirit of incompleteness and conviviality without necessarily identifying certain labels and certain stories as outdated and superficial. Such practical activities that go beyond rhetoric to action is what is required if Africa is to achieve ‘liberatory promise of the anticolonial nationalist eras’. (15)
- We affirm de Vries assertion that “incompleteness and conviviality are part of a rich African intellectual tradition to help articulate opportunities for a transformative research agenda that helps us understand our world, and its crises, better.” (3) However, we quickly ask: might incompleteness and conviviality be a different way of expressing Ubuntu or might Ubuntu be a broader expression of incompleteness and conviviality? For now, the big challenge remains how to translate African theoretical perspectives (science and innovation) on ethics, of whatever sphere but especially global health ethics, into practice. We end this review with an acknowledgement and recognition of the practical work that de Vries is directing in the Ethics Lab at the University of Cape Town. This is exactly what centering Africa as the context and driver for global health ethics is about. We salute you, Jantina!    

[1] The Circle of Concerned African Women Theologians (Circle), of which this reviewer is a member, is a Pan-African ecumenical organization whose mission is to provide space for African women “to undertake research, writing and publishing on African issues from women’s perspectives.” Circle of Concerned African Woman Theologians

Does the article adequately reference differing views and opinions?

Yes

Are all factual statements correct, and are statements and arguments made adequately supported by citations?

Yes

Is the Open Letter written in accessible language?

Yes

Where applicable, are recommendations and next steps explained clearly for others to follow?

Yes

Is the rationale for the Open Letter provided in sufficient detail?

Yes

Reviewer Expertise:

Health research ethics; religion/spirituality and health; gender and wellbeing

I confirm that I have read this submission and believe that I have an appropriate level of expertise to confirm that it is of an acceptable scientific standard.

## References

[1] : Towards a common definition of global health. *Lancet* .2009;373(9679) : 10.1016/S0140-6736(09)60332-9 1993-5 10.1016/S0140-6736(09)60332-9 19493564 PMC9905260

[2] : Defining global health: findings from a systematic review and thematic analysis of the literature. *BMJ Glob Health* .2021;6(6) : 10.1136/bmjgh-2021-005292 10.1136/bmjgh-2021-005292 34083243 PMC8183196

[3] De Vries J. Centering Africa as context and driver for Global Health Ethics: incompleteness, conviviality and the limits of Ubuntu. *Wellcome Open Research 2024; 9:371* . 10.12688/wellcomeopenres.22508 PMC1250492941069415

[4] Nyamnjoh, F. B. Incompleteness, mobility and conviviality. *Bamenda, Cameroon Langaa RPCIG, Langaa, 2023* .

[5] De Vries, J. And Bull, S. “Reflecting on the ethical challenges of conducting genomic research in Africa” in Annual Review of Genomics and Human Genetics, 2019; 20: 431-451.

[6] Benatar, S. Global Health and Global Health Ethics: Critical Reflections on the Role of power and inequality”. *Global Public Health, 2019; 1492: 185-198.* .

[7] Hyder, A. A. and Merritt, M. W. Defining Global health ethics: a transdisciplinary challenge.”. *Global Health Action, 2019; 12(1), 1627482.* .

[8] Ong’era F. Mogaka, Jenell Stewart and Elizabeth Bukusi. Why and for whom are we decolonizing global health. *The Lancet Global Health. October 2021; 9(10):E1359-E1360* . 10.1016/S2214-109X(21)00317-X 34534477

[9] Abimbola, S., & Pai, M. Will global health survive its decolonisation? The Lancet, (2020); 396(10263), 1627-1628. 10.1016/S0140-6736(20)32417-X33220735

[10] Affun-Adegbulu C, Adegbulu O Decolonising Global (Public) Health from Western Universalism to global Universalism. BMJ Global Health, (August 2020); 5 (8):002947. doi:10.1136/bmjgh-2020-002947. PMC 7443258. PMID 32819916. S2CID 221220715. 10.1136/bmjgh-2020-002947PMC744325832819916

[11] Anane-Binfoh NA, Flaherty KE, Zakariah AN, Nelson EJ, Becker TK, Afaa TJ. Barriers to decolonizing global health: identification of research challenges facing investigators residing in low- and middle-income countries. Glob Health Sci Pract. 2024;12(1): e2300269. https://doi.org/10.9745/GHSP-D-23-00269.10.9745/GHSP-D-23-00269PMC1090655038242635

[12] Kwete X, Tang K, Chen L, Ren R, Chen Q, Wu Z, et al. “Decolonizing global health: what should be the target of this movement and where does it lead us? Global Health Research and Policy. January 2022, 7 (1):3. doi:10.1186/s41256-022-00237-3. PMC 8784247. PMID 35067229."Decolonizing global health: what should be the target of this movement and where does it lead us?". 10.1186/s41256-022-00237-3PMC878424735067229

[13] Rasheed M. A. Navigating the violent process of decolonizing global health research: a guideline. The Lancet Global Health. December 2021; (9)12: e1640-e1641 doi:10.1016/S2214-109X(21)00440-X. PMID 34798014. S2CID 244286291. 10.1016/S2214-109X(21)00440-X34798014

[14] Desmond Tutu. Obedience to God University of California press, 2010. p. 279 https://publishing.cdlib.org/ucpressebooks/view?docId.

[15] Mama A: Is It ethical to study Africa? preliminary thoughts on scholarship and freedom. Afr Stud Rev. 2007; 50(1):1.
